# Efficient Cellular Release of Rift Valley Fever Virus Requires Genomic RNA

**DOI:** 10.1371/journal.pone.0018070

**Published:** 2011-03-21

**Authors:** Mary E. Piper, Dorothy R. Sorenson, Sonja R. Gerrard

**Affiliations:** 1 Cellular and Molecular Biology Program, University of Michigan, Ann Arbor, Michigan, United States of America; 2 Microscopy and Image-Analysis Laboratory, University of Michigan, Ann Arbor, Michigan, United States of America; 3 Department of Epidemiology, University of Michigan, Ann Arbor, Michigan, United States of America; Duke University Medical Center, United States of America

## Abstract

The Rift Valley fever virus is responsible for periodic, explosive epizootics throughout sub-Saharan Africa. The development of therapeutics targeting this virus is difficult due to a limited understanding of the viral replicative cycle. Utilizing a virus-like particle system, we have established roles for each of the viral structural components in assembly, release, and virus infectivity. The envelope glycoprotein, Gn, was discovered to be necessary and sufficient for packaging of the genome, nucleocapsid protein and the RNA-dependent RNA polymerase into virus particles. Additionally, packaging of the genome was found to be necessary for the efficient release of particles, revealing a novel mechanism for the efficient generation of infectious virus. Our results identify possible conserved targets for development of anti-phlebovirus therapies.

## Introduction

Rift Valley fever virus (RVFV) is an aerosol- and mosquito-borne virus endemic to sub-Saharan Africa [1]. RVFV causes periodic, explosive epizootics, affecting livestock and humans [1]. Sheep and cattle are particularly susceptible to the virus, with abortion rates approaching 100% and high mortality rates among young animals [2]. Most humans infected with RVFV have a flu-like illness [1]. However, a small percentage of cases are more severe and include manifestations such as hemorrhagic disease and encephalitis [3], [4], [5]. Despite the severity of the disease to the economy and human health, there are no USDA or FDA-approved therapeutic or prophylactic treatments. A better understanding of the RVFV replication cycle may lead to the identification of novel therapeutic targets. In this study, we have identified roles for each of the viral structural components in the assembly and release of RVFV and have identified a potential conserved target for therapeutic development.

RVFV is a segmented, negative-sense RNA virus belonging to the family *Bunyaviridae*, genus *Phlebovirus*. The 12 kilobase genome is comprised of three segments termed L, M and S, which encode for the RNA-dependent RNA polymerase (RdRp), envelope glycoproteins (Gn/Gc) and nucleocapsid protein (N), respectively [1]. The S and M segments also encode nonstructural proteins known as NSs and NSm, however these proteins are dispensable for RVFV replication in cell culture [6], [7], [8]. Upon entry into host cells, the encapsidated genome and RdRp are released into the cytoplasm where transcription and replication of the viral genome occurs [1]. RdRp acts as both transcriptase and replicase [9], but requires N for both activities [10]. RdRp and N do not contain signal peptides, and are presumably translated on cytoplasmic ribosomes. The glycoproteins enter the secretory pathway as a precursor polyprotein, which is cleaved by signal peptidase to yield mature Gn and Gc [11]. Gn and Gc form a complex and localize in steady-state to the Golgi apparatus, the site of virus assembly, due to a localization signal on Gn [11], [12], [13]. It is not known how the encapsidated genome and RdRp are recruited to the Golgi apparatus for virus assembly or which viral components are involved in the cellular release of virus.

Utilizing a Rift Valley fever virus-like particle (RVF-VLP) system, we have determined that encapsidated genome acts as the primary stimulus for RVFV release from the cell. The driving of virus release by encapsidated genome is an elegant mechanism for ensuring that infectious particles are the dominant specie released from cells. We demonstrate that Gn is necessary and sufficient for packaging of the RdRp and N. Furthermore, we show that distinct regions of the Gn cytosolic tail are required for binding RdRp and N. These data provide the most complete description of RVFV assembly and release to date, and suggest novel targets of the development of anti-phlebovirus drugs.

## Materials and Methods

### Plasmid Constructs

All plasmids were generated using standard molecular cloning techniques and were confirmed by sequencing. The constructs pTrRVFV-SΔNSs::GFP, pN-Amp, pRdRp-Amp, pGn/Gc-Amp, and pGnK48Stop-Amp have been described previously [6], [12]. The minigenome, pSTrRVFV-SΔNΔNSs::hRLuc, was derived from pTrRVFV-SΔNSs::GFP by replacing the GFP gene with a humanized renilla luciferase gene (RLuc), then deleting a 237 nucleotide SmaI fragment of the N gene. The expression constructs for N and RdRp were generated through cloning the open reading frames into pVAX1 (Invitrogen) using the HindIII/EcoRI and BamHI/NotI sites, respectively. The open reading frames from pGn/Gc-Amp and pGnK48Stop-Amp were cloned into pVAX1 using BamHI and EcoRI sites, generating pGn/Gc, pGc, pGn, and pGnK48. The expression plasmid, pGcW1, was generated by site-directed mutagenesis of Trp1189 to a stop codon in pGc, thus deleting the entire predicted cytoplasmic tail. Site-directed mutagenesis of pRdRp generated the catalytic domain RdRp mutant alleles pRdRp*^cat1^* and pRdRp*^cat2^*, which were mutated to Ala at residues Asp1134 and Ser1132, respectively.

### Cells and virus

BSR-T7/5 cells were a generous gift of Dr. K. Conzelmann (Max-von Pettenkofer-Institut, Munchen, Germany). The BSR-T7/5 clonal cell line was generated through limiting dilution of the BSR-T7 cells. The cells were grown in Dulbecco's Modified Eagle Medium (Invitrogen) supplemented with 10% fetal calf serum, and 1 mg/mL Geneticin. RVFV ZH548 MP12 vaccine strain was a generous gift of Dr. R. Tesh (World Reference Center of Emerging Viruses and Arboviruses).

### Antibodies

Hybridomas that secrete neutralizing monoclonal antibodies recognizing Gn and Gc (R1-4D4-1-1 and R5-3G2-1A) were a generous gift of Dr. G. Ludwig (USAMRIID). Polyclonal antibodies that were generated against RVFV in mice were a generous gift of Dr. P. Rollin (CDC). The N-terminal 150 amino acids of the RdRp and full-length N were expressed with N-terminal histidine tags and purified under denaturing conditions on Ni-NTA agarose columns (Qiagen Inc.). RdRp and N polyclonal antibodies were generated in rabbits using these purified proteins as antigens (Harlan Laboratories). Monoclonal antibodies recognizing GS-28 and β-COP were purchased from Transduction Labs and ABR, respectively. Horseradish peroxidase-conjugated secondary antibodies, goat anti-rabbit and goat anti-mouse, were acquired from Amersham and MP Biomedical, respectively. AlexaFluor 488-labelled goat anti-rabbit and AlexaFluor 594-labelled goat anti-mouse were purchased from Invitrogen.

### Virus-like particle production

BSR-T7/5 cells were plated at a density of 1.2×10^6^ cells/plate. After 24 h, cells were transfected using 2 µL TransIT LT1 (Mirus Corporation)/µg DNA and plasmids in the ratio 6.0 µg minigenome: 6.0 µg pN: 6.0 µg pRdRp: 3.0 µg pGn: 3.0 µg pGc/10 cm plate. The amount of plasmid transfected was scaled to the number of cells. The media was changed 24 h post-transfection. After 48 h post-transfection, the RVF-VLPs were harvested from the media, then clarified by low-speed centrifugation (300 rcf for 10 min at 4°C) to remove cellular debris. The transfected cells were analyzed by RLuc assay (Promega) in order to verify that RLuc activity levels were similar across experimental attempts.

### Virus-like particle infections

BSR-T7/5 cells were used as target cells for RVF-VLP infections. These cells were either not transfected or transfected using 2 µL TransIT LT1 (Mirus Corporation)/µg DNA and 0.25 µg pRdRp and 0.25 µg pN per well of a 24-well plate. Transfected cells were infected with RVF-VLPs 24 h post-transfection. RVF-VLP-infected target cells were harvested 24 h post-infection and were analyzed for RLuc activity. The raw luciferase units (RLU)/mL of RVF-VLPs added to target cells was calculated for three or more separate experiments. The log of the average RLU/mL was calculated for analysis by Independent T-Test (SPSS Statistical Package 14.0), and compared to the negative control (-Gn/-Gc).

### High-speed ultracentrifugation of RVF-VLPs

Clarified samples containing RVF-VLPs were subjected to high-speed ultracentrifugation at 82,000 g for 16 h using either a SW-28 or SW50.1 rotor, depending on sample size. The supernatant was decanted and the pellet was resuspended in Laemmli sample buffer, citrate buffer (10 mM citrate, pH 6.4) or 0.1 M Sorensen phosphate buffer, depending on experiment.

### Immune precipitation of RVF-VLPs

Mouse monoclonal antibodies recognizing either Gn (R1-4D4-1-1) or Gc (R5-3G2-1A) were conjugated to Dynal magnetic beads (Invitrogen) by incubating overnight at 4°C. The antibody-coated beads were incubated overnight at 4°C with RVFV or RVF-VLPs, then washed with Wash Buffer (10 mM Tris, 5 mM MgCl_2_, and 100 mM NaCl, pH 7.8), and resuspended in 1X Laemmli sample buffer for analysis by immunoblot. To prevent variation between conditions, generation of RVF-VLPs, immune precipitation, and immunoblotting were performed for all conditions at the same time. The representative immunoblots in the figures are from a single immunoblot split into the different figures. Therefore, each figure displays the same positive (WT) and negative (-Gn/-Gc) controls for comparison. The extensive experiments were performed multiple times, but only an immunoblot from a single experiment is shown.

### Transmission electron microscopy

RVFV and RVF-VLPs were pelleted by high-speed ultracentrifugation and resuspended in 0.1 M Sorensen phosphate buffer, and distributed onto a carbon-coated grid. The particles were fixed with 2.5% glutaraldehyde in Sorensen phosphate buffer and negative stained with aqueous 1% uranyl acetate, which was performed by the Microscopy Imaging Laboratory (University of Michigan). The particles were viewed on a Philips CM100 transmission electron microscope at 60 KV. Images were recorded digitally using a Hamamatsu ORCA-HR digital camera system, which was operated using AMT software (Advanced Microscopy Techniques Corp., Danvers, MA). The sizes of RVFV and RVF-VLPs were measured using ImageJ software (NIH). The diameter of particles was measured outer membrane to outer membrane for a minimum of 10 particles per condition.

### RT-PCR detection of genomic RNA in RVF-VLPs

RVF-VLPs were generated as described above, except that at 24 h the media was replaced with fresh media that contained 90 U/mL Benzonase (Novagen). At 48 h RVF-VLPs were harvested from 4 mL of media by high-speed ultracentrifuation. The pelleted material was resuspended in PBS and the RNA isolated by extraction with phenol/chloroform followed by ethanol precipitation. The pellet was resuspended in 20 µL citrate buffer. cDNA was generated from 4 µL of RNA using M-MuLV reverse transcriptase (New England BioLabs) and primers that recognize the 5′ and 3′ termini of the genomic RNA (ACACAAAGCTCCCTAGAGATAC and AAGCACTAGGGGGTCTTTGTGT). The cDNA was amplified (29 cycles) using Phusion polymerase (New England BioLabs) and primers that anneal within N (CATGAGAAGAGGAGAGAATTCT) and RLUC (ACGATGGCCTTGATCTTGTC). The expected RT-PCR product size is 773 nucleotides and includes the intergenic region of the genome.

### Chemical cross-linking and co-immunoprecipitation of Gn and N

BSR-T7/5 cells were grown in 12-well plates and transfected with the plasmids indicated in the figure. The amount of DNA transfected was held constant by addition of empty vector (pVAX1) when necessary. At 48 h post-transfection the media was removed and the cells were washed with PBS. The cells were then exposed to 250 µM Dithiobis[succinimidyl] propionate (Pierce) in PBS for 30 min at room temperature. The cross-linking solution was removed at the end of the incubation and replaced with 400 µL of 5X RIPA buffer (250 mM Tris-HCl, pH 7.5, 0.75 M NaCl, 5 mM EDTA, 5% Triton X-100, 5% sodium deoxycholate, 1% SDS). The samples were then diluted to 1X RIPA by the addition of water. Insoluble material was removed by centrifugation at 16,000 g for 5 min. Mouse monoclonal antibodies recognizing Gn (R1-4D4-1-1) were conjugated to protein G agarose (Pierce) by incubating overnight at 4°C. The samples were incubated with the antibody-coated beads for 1 h at 4°C then washed three times with 1X RIPA buffer. The final wash was removed and the beads were resuspended in Laemmli sample buffer with 10% β-mercaptoethanol. The samples were analyzed by immunoblot using rabbit anti-Gn and anti-N antibodies.

### Immunofluorescence

BSR-T7/5 cells were plated on glass coverslips at 5.0×10^4^ cells/well of a 24-well plate. After 24 h, the cells were transfected using 2 µL TransIT/µg DNA. The cells were fixed 24 h post-transfection with 4% paraformaldehyde in phosphate-buffered saline (PBS), then permeabilized using 0.2% Triton X-100 in PBS with 1% bovine serum albumin. Mouse monoclonal antibodies recognizing Gn and Gc and rabbit polyclonal antibodies recognizing the RdRp and N were used as primary antibodies, while AlexaFluor488-labelled goat anti-rabbit and AlexaFluor 594-labelled goat anti-mouse were used as secondary antibodies (Invitrogen). Fluorescence visualization and imaging were performed using an Olympus 51-X fluorescent light microscope at the Microscopy Imaging Laboratory (University of Michigan). Cells with clear signals for both red (594 nm) and green channels (488 nm) were counted, then, analyzed for co-localization. Positive co-localization was defined as the RdRp exhibiting a focus of intense staining corresponding to the Golgi/glycoprotein signal. Diffuse cytoplasmic staining and small puncta in the cytoplasm were not counted for positive co-localization.

### Efficiency of RVF-VLP Cellular Release

Efficiency of cellular release was determined through quantitation of Gn/Gc levels in the cell lysates and within the RVF-VLPs. RVF-VLPs were purified through high-speed ultracentrifugation or immune precipitation. Both methods generated similar results for the release efficiencies, therefore immunoblots from both types of purification were combined to calculate the average release efficiencies with standard deviation and to perform the statistics.

Immunoblots were scanned on a PhosphoImager and analyzed using ImageQuant 5.2 (Molecular Dynamics) to determine the signal intensity (volume). Glycoprotein signal volume from the cell lysates was divided by background volume to attain the normalized glycoprotein expression levels in the cell lysates. The glycoprotein signal volume for RVF-VLPs was divided by the normalized glycoprotein signal from the corresponding cell lysate. Normalizing the glycoprotein signal for RVF-VLPs had little to no effect on the calculated release efficiencies for conditions lacking genome, N, RdRp or with the RdRp*^cat1^* allele since glycoprotein expression levels were similar across these conditions. However, the immunoblot signals for GnK48 and GcW1 alleles were lower (∼25–50%, depending on experiment) than for their wild-type counterparts making it necessary to normalize for input. Release efficiencies were calculated as a percentage of the WT condition. Statistics were performed for the comparison of glycoprotein expression levels from experiments performed in triplicate using One Sample T-Tests (SPSS Statistical Package 14.0).

## Results

### RVFV and RVF-VLPs have similar morphology and protein content

**Figure 1:**
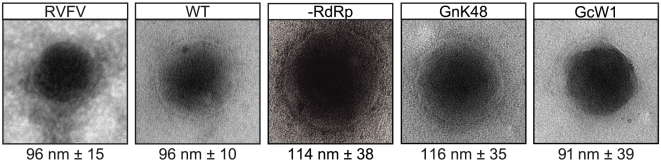
RVFV and RVF-VLPs have similar morphology. RVFV and RVF-VLPs were harvested by ultracentrifugation and analyzed by transmission electron microscopy with negative staining. The particle sizes were measured, and the values listed are the mean sizes of particles with standard deviation.

**Figure 2:**
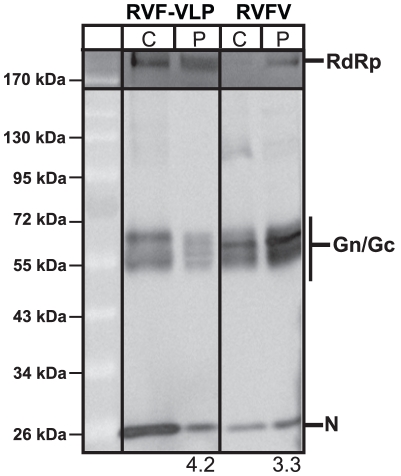
RVFV and RVF-VLPs have similar protein composition. Lysates from transfected (RVF-VLP) or RVFV-infected cells (C), and pelleted particles (P) were analyzed by immunoblot. The numbers below the immunoblots indicate the ratio of glycoprotein to N signal.

A T7 RNA polymerase-dependent system was used for the efficient generation of RVF-VLPs [14]. Briefly, RVF-VLPs were produced by expression of an S segment-based minigenome (pSTrRVFV-SΔNΔNSs::hRLuc), N, RdRp, Gn, and Gc in BSR-T7/5 cells. The minigenome contains a humanized renilla luciferase (RLuc) gene in place of the NSs ORF and an internal deletion in the N gene that prevents expression of N [14]. RVFV and RVF-VLPs were harvested by ultracentrifugation and analyzed for particle morphology by transmission electron (Fig. 1) and protein composition by immunoblot (Fig. 2). RVFV and the RVF-VLPs exhibited similar size and morphology by transmission electron microscopy. All of the viral proteins were detected in the cell lysates (C) and pelleted material (P) for RVFV and the RVF-VLPs (Fig. 2). Similar ratios of glycoprotein to N were found in RVFV and RVF-VLPs (Fig. 2), although the glycoprotein to RdRp ratio was higher for RVF-VLPs (2.3 versus 6.0). The latter result was not surprising given that much more RdRp was found in transfected versus infected cells. There appears to be some differences with respect to the species of glycoproteins present in RVFV and RVF-VLP preparations in both the cell lysate and pelleted material (Fig. 2). These differences may reflect the fact that our glycoprotein expression construct does not include the NSm region of the M segment, a region that is dispensable for virus maturation, replication and infection [6], [8]. The envelope glycoproteins are synthesized as an N-terminal nested set that yields at least two mature glycoproteins containing the NSm region [11], [15], [16]. In addition to similarities in particle morphology and protein composition, RVFV and the RVF-VLPs are antigenically indistinguishable and respond similarly to inhibitor compounds [14]. All of our data suggest the RVF-VLPs function similar to virus and will be useful in dissecting steps of the RVFV replication cycle.

### Gn recruits RdRp from the cytoplasm

**Figure 3:**
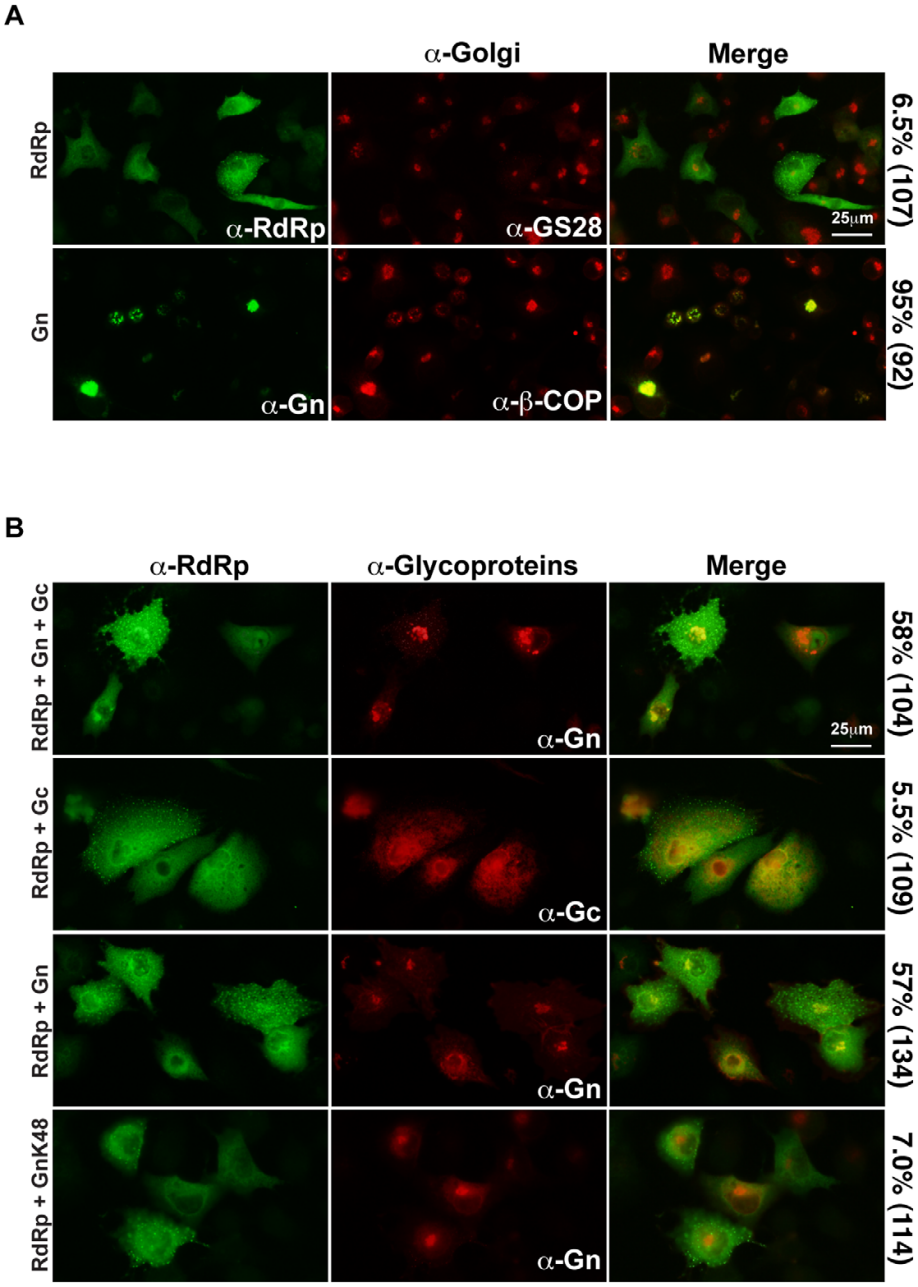
Gn recruits RdRp. **A.** BSR-T7/5 cells were transfected with pRdRp or pGn, and the proteins were visualized with anti-RdRp and anti-Gn, respectively (green channel). Cellular resident Golgi apparatus proteins, GS-28 or β-COP were also labeled (red channel). Percentage of cells displaying co-localization of viral proteins with resident Golgi proteins is indicated with the number of cells counted in parentheses. **B.** BSR-T7/5 cells were transfected with pRdRp and either pGn/pGc, pGc, pGn, or pGnK48. Cells were incubated with anti-RdRp (green channel) and anti-Gn or anti-Gc (red channel), and then analyzed by immunofluorescence microscopy. Percentage of cells displaying co-localization of RdRp with Gn or Gc is indicated with the number of cells counted in parentheses.

Replication and transcription of the viral genome by RdRp occurs in the cytoplasm and assembly of virus particles takes place at the Golgi apparatus [1]. We investigated the localization of RdRp in the absence of other viral proteins. It is believed that all bunyavirus RdRp are translated on free ribosomes in the cytoplasm [1], however the localization of wild-type RVFV RdRp had not been determined previously. When expressed in the absence of other viral proteins, RdRp was found distributed diffusely throughout the cytoplasm and did not co-localize with the resident Golgi protein, GS-28 (Fig. 3A). By contrast, Gn co-localized with the resident Golgi protein β-COP (Fig. 3A), in agreement with previously published reports [12]. The envelope glycoproteins are presumably responsible for recruitment of RdRp to the site of virus assembly, the Golgi apparatus. We tested this hypothesis by co-expressing RdRp with the glycoproteins then determining if the cytoplasmic localization of RdRp was altered. Gn and Gc are integral membrane proteins that are expressed as a polyprotein precursor [11], [15]. The polyprotein is cleaved by signal peptidase, generating mature Gn and Gc [11]. It is believed that mature Gn retains the signal peptide of Gc [11], [16]. Gn and Gc form a heteromeric complex that localizes in steady-state to the Golgi apparatus [11], [12]. Expression of the glycoproteins along with RdRp resulted in localization of RdRp to a focus of intense staining co-localizing with Gn (Fig. 3B), indicating that one or both glycoproteins is necessary for recruitment of RdRp. When Gc was co-expressed with RdRp, the cytoplasmic localization of RdRp was not altered (Fig. 3B). By contrast, co-expression of Gn was sufficient to alter the localization of RdRp (Fig. 3B). A portion of the RdRp co-localized with Gn in 57% of cells, indicating that Gn is necessary and sufficient for the recruitment of RdRp.

The domain within Gn that is responsible for the recruitment of RdRp was identified using a Gn allele that lacks the last 40 amino acids of the cytoplasmic tail and the Gc signal peptide (GnK48). Although GnK48 localizes properly to the Golgi apparatus [12], it was unable to recruit RdRp (Fig. 3B). Therefore, the last 40 amino acids of the Gn cytoplasmic tail and/or the Gc signal peptide is necessary for the recruitment of RdRp.

### Generation of infectious RVF-VLPs requires packaging of a catalytically active RdRp

**Figure 4:**
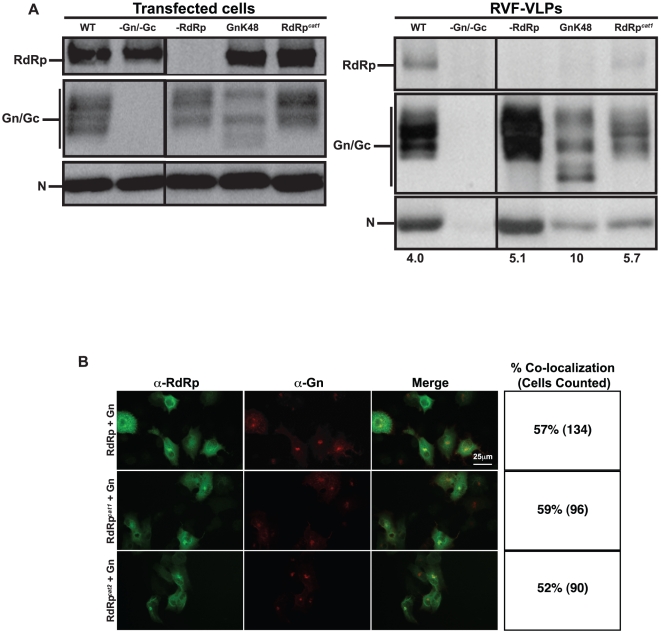
Packaged, catalytically active RdRp is necessary for an early event in the replicative cycle. **A.** BSR-T7/5 cells were transfected with genome and all of the structural proteins (WT), or one or more of the components was replaced with an equivalent amount of empty vector (-Gn/Gc and –RdRp) or with plasmids expressing mutant alleles of Gn or RdRp (GnK48 or pRdRp*^cat1^*). Transfected cells were analyzed for protein expression by immunoblot. RVF-VLPs were immune precipitated from the clarified media from transfected cells and analyzed by immunoblot. The numbers below the immunoblots indicate the ratio of glycoprotein to N signal. **B.** BSR-T7/5 cells were transfected with pGn and either pRdRp or RdRp catalytic domain mutants, pRdRp*^cat1^* or pRdRp*^cat2^*. Cells were incubated with anti-Gn (red channel) and anti-RdRp (green channel), and then analyzed by immunofluorescence microscopy. Percentage of cells displaying co-localization of RdRp alleles with Gn is indicated with the number of cells counted in parentheses.

Packaging of RdRp into virus particles is necessary for RVFV to produce progeny in infected cells. However, it is not known whether RdRp is necessary for efficient production of virus particles. This question was addressed by determining if RVF-VLPs could be produced when RdRp was absent or when the Gn mutant that fails to recruit RdRp (GnK48) was expressed. BSR-T7/5 cells were transfected with minigenome, pN, pRdRp, pGn, and pGc or one or more of the components were replaced with an equivalent amount of empty vector or pGnK48. RVF-VLPs were visualized by transmission electron microscopy and immune precipitated RVF-VLPs were analyzed for protein composition by immunoblot. Only WT, –RdRp and GnK48 conditions generated quantities of RVF-VLPs that were sufficient for accurate estimates of RVF-VLP size. RVF-VLPs made in the absence of RdRp or expressing GnK48 did not display gross differences in morphology as compared to WT (Fig. 1), indicating that RdRp is not required for generation of particles. As expected, RdRp signal was present in RVF-VLPs when all components were expressed (WT) and absent when RdRp was not expressed (-RdRp) (Fig. 4A). When the GnK48 allele was expressed, almost no RdRp was found in RVF-VLPs (Fig. 4A). This result is in agreement with the co-expression results (Fig. 3B) and confirms that GnK48 is unable to recruit RdRp. Although RdRp is not required for RVF-VLP production, particles that lack RdRp (-RdRp and GnK48) are ∼20% larger than WT and display greater size variability (Fig. 1), indicating that loss of RdRp is not innocuous. Additionally, there appears to be some subtle differences in glycoprotein species found within cells, and possibly within RVF-VLPs, when RdRp is absent (Fig. 4A). We do not know the molecular nature of these differences, however since RdRp and Gn interact, it would not be surprising if RdRp altered the trafficking and/or post-translational modification of Gn. Glycoprotein to N ratios for WT and no RdRp (-RdRp) conditions were similar, indicating that loss of RdRp did not adversely impact packaging of N. We then determined whether a catalytically active RdRp is required for interaction with Gn. Neither catalytic domain RdRp mutant has measurable catalytic activity (data not shown). Similar to wild type RdRp, RdRp*^cat1^* and RdRp*^cat2^* co-localized with Gn, indicating that catalytic activity is not required for this interaction (Fig. 4B). These results are supported by our immunoblot results showing that RdRp*^cat1^* is packaged into RVF-VLPs (Fig. 4A).

We next investigated whether catalytically active RdRp expressed in target cells (*trans* expression) could rescue infectivity of RdRp-deficient or RdRp*^cat1^* RVF-VLPs. Wild-type RVF-VLPs were capable of infecting untransfected target cells and had RLuc activity that was 700-fold above background (Table 1). Transcription of the RLuc reporter in target cells could be enhanced through expression of a catalytically active RdRp in *trans*, increasing the RLuc signal to 4,000-fold background levels (Table 1). Expression of RdRp*^cat1^* in target cells did not enhance RLuc signal as compared to untransfected target cells (Table 1), indicating that the observed signal enhancement by wild-type RdRp is due to catalytic activity and not cooperativity. By contrast, RVF-VLPs packaging RdRp*^cat1^* or RVF-VLPs lacking the RdRp (-RdRp) could not be complemented in *trans* with an active RdRp (Table 1). Wild-type RVF-VLPs were the only RVF-VLPs to generate a significant RLuc signal as compared to background (-Gn/-Gc) (Table 1). These results indicate that catalytically active RdRp must be packaged in the RVF-VLP in order for the *trans* RdRp to transcribe the genomic RNA (Table 1).

**Table 1:** RdRp and N in *trans* fail to rescue RdRp-deficient RVF-VLPs.

Sample	Untransfected		RdRp*^cat1^*/N		RdRp/N	
	AverageLog (RLU/mL)	Std. Dev.	AverageLog (RLU/mL)	Std. Dev.	AverageLog (RLU/mL)	Std. Dev.
WT	6.61*	0.782	6.51*	1.10	8.15*	0.471
-Gn/-Gc	3.76	0.296	3.42	0.325	4.47	0.515
-RdRp	n.d.		3.53	0.319	4.60	1.00
GnK48	n.d.		3.56	0.317	4.78	0.484
RdRp*^cat1^*	n.d.		3.60	0.231	4.22	0.815

*Values are significantly different from -Gn/-Gc, p<0.005.

n.d.; not determined.

### N is packaged into virions by Gn

**Figure 5:**
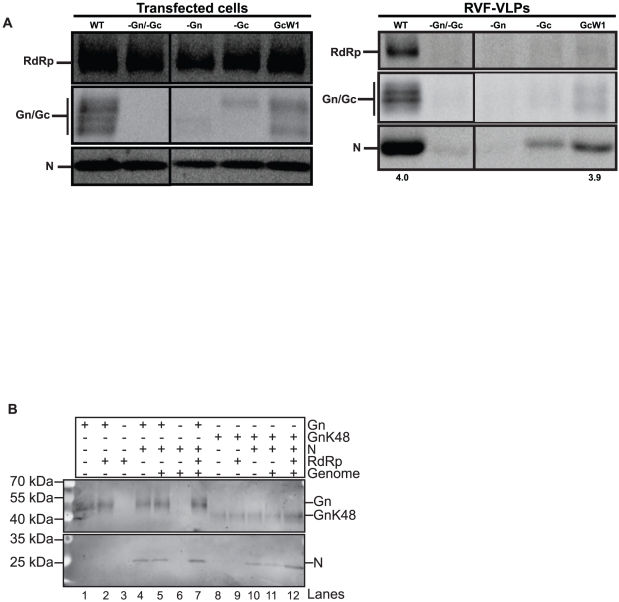
Gn packages N. **A.** BSR-T7/5 cells were transfected with genome and all of the structural proteins (WT), or one or more of the components was replaced with an equivalent amount of empty vector, (-Gn/-Gc, -Gn, or -Gc) or a plasmid expressing an allele of Gc that lacks the entire cytoplasmic tail (GcW1). Transfected cells were analyzed for protein expression by immunoblot. RVF-VLPs were immune precipitated from the clarified media from transfected cells and analyzed by immunoblot. The numbers below the immunoblots indicate the ratio of glycoprotein to N signal. **B**. BSR-T7/5 cells were transfected with the indicated plasmids and proteins were cross-linked at 48 h post-transfection. Mouse monoclonal anti-Gn antibodies were used to immune-precipitate Gn containing complexes. The cross-links were cleaved and then Gn-containing complexes were identified by immunoblot using rabbit anti-Gn or rabbit anti-N antibodies.

After transcription and replication of the viral genome in the cytoplasm, we hypothesized that encapsidated genome and RdRp were recruited as a complex to the Golgi apparatus for assembly through interaction between RdRp and Gn. However, we discovered that N could be packaged into RVF-VLPs lacking RdRp (Fig. 4A, -RdRp and GnK48). This result indicates that N and RdRp can be packaged independently. Phlebovirus N localizes to the cytoplasm when expressed alone (data not shown), similar to N of tomato spotted wilt virus (*Tospovirus* genus) [17] and La Crosse virus (*Orthobunyavirus* genus) [18], but in contrast to Hantaan and Black Creek Canal viruses N (*Hantavirus* genus) [19], [20]. Phlebovirus N presumably interact with one or both of the envelope glycoproteins in order to be assembled into virions. The GnK48 allele was able to package N (Fig. 4A), which indicates that the last 40 amino acids of the 70 amino acid Gn cytoplasmic tail and the Gc signal peptide are not required for packaging of N. Accordingly, Gc and/or the first 30 amino acids of the Gn cytoplasmic tail appear necessary for its packaging. To determine whether Gn or Gc is involved in N packaging, we transfected cells with all viral components or equivalent amounts of plasmid encoding the GcW1 allele or empty vector. GcW1 has a premature stop codon at Trp1189, which deletes the predicted Gc cytoplasmic domain in its entirety. Particles lacking Gn or Gc, or containing GcW1, were analyzed for morphology and protein content. Only WT and GcW1 produced particles efficiently, and thus particle sizes were only measured for these two conditions (Fig. 1). Deletion of the cytoplasmic tail of Gc (GcW1) had no effect on average particle size or morphology as compared to WT RVF-VLPs, however there was more size variation (Fig. 1). The level of glycoproteins expressed in transfected cells varied by experimental condition (Fig. 5A). Co-expression of full-length Gn and Gc was required for high-level expression of each glycoprotein. Previous studies with the Bunyamwera virus (*Orthobunyavirus* genus) identified a chaperone-like role for Gn in the folding of Gc and a requirement for the Gc ectodomain for efficient Golgi trafficking of Gn [21], [22]. Therefore, finding reduced levels of Gc and Gn when either was expressed alone was not surprising. The average glycoprotein signal within RVF-VLPs generated with Gn or Gc alone was near background levels (Fig. 5A). Interestingly, N was still packaged into RVF-VLPs that lack Gc (Fig. 5A). No N was found in RVF-VLPs that lacked Gn (Fig. 5A), however since Gc levels are at background it is not possible to interpret this result. When Gn was expressed with GcW1, both N and RdRp were packaged into RVF-VLPs. The ratio of glycoprotein to N for GcW1 and WT conditions was similar, supporting the view that the cytosolic tail of Gc is dispensable for packaging of N. Consistent with the immunoblot results (Fig. 5A), GcW1 RVF-VLPs were infectious and yielded RLuc activity that was significantly above background levels in target cells complemented in *trans* with active RdRp and N (Table 2).

**Table 2 pone-0018070-t002:** Gc cytosolic tail is dispensable for infectivity.

Sample	AverageLog (RLU/mL)	Std. Dev.Log (RLU/mL)
WT	8.15*	0.471
-Gn/-Gc	4.47	0.515
-Gn	4.52	0.672
-Gc	4.82	1.10
GcW1	5.86*	0.748

*Values are significantly different from -Gn/-Gc, p<0.01.

We then investigated whether Gn and N interaction is modulated by genome or RdRp. BSR-T7/5 cells were transfected with minigenome, pN, pRdRp, pGn, or one or more of the components were replaced with an equivalent amount of empty vector or pGnK48. At 48 h post-transfection, cellular proteins were cross-linked with a thiol cleavable cross-linker (Dithiobis[succinimidyl] propionate), then Gn containing complexes were immune precipitated with monoclonal anti-Gn antibodies. The cross-linker was cleaved with β-mercaptoethanol and Gn and N were analyzed by immunoblot (Fig. 5B). Presence of the RdRp in these complexes was also analyzed, however the RdRp signal in our positive control was not sufficient for reliable interpretation of the results (data not shown). Gn-N interaction was independent of RdRp (Fig. 5B, compare lanes 5 and 7), consistent with our ability to form RVF-VLPs in the absence of RdRp (Fig. 1 and Table 3). We were able to pull down N with Gn whether or not genome was present (Fig. 5B, compare lanes 4 with 7). The GnK48 mutant is also able to interact with N (Fig. 5B, lanes 10-12), indicating that sequence required for N recruitment is within the first 30 amino acids of the Gn cytosolic tail. These results are in agreement with the results for RVF-VLPs generated with GnK48 (Fig. 4A), in that GnK48 can bind N but fails to bind RdRp. While genomic RNA is not required for Gn-N interaction (Fig. 5B, compare lanes 4 and 5), the interaction may still be RNA-dependent, since N has been shown to be a non-specific single-stranded RNA binding protein [23]. In the case of bacterially expressed N, ∼90% is bound to RNA [23] and we expect that the same is true of N expressed in mammalian cells.

**Table 3:** Encapsidated genome required for efficient cellular release.

Sample	% Efficiency	% Std. Dev.
-RNPs	14.2	‡
-N	15.6*	8.8
-Genome	18.1*	3.7
-RdRp	169.5	71.8
GnK48	49.9†	13.3
RdRp*^cat1^*	37.1‡	16.5
-Gn	Δ	
-Gc	Δ	
GcW1	56.9	20.4

*Values are significantly different from WT RVF-VLP release efficiency, p<0.005.

†Value is significantly different from WT RVF-VLPs release efficiency, p<0.05.

ΔYields were below the limit of detection.

‡Insufficient number of replicates for std. dev. and p-value calculations.

### Genome triggers virus release

**Figure 6:**
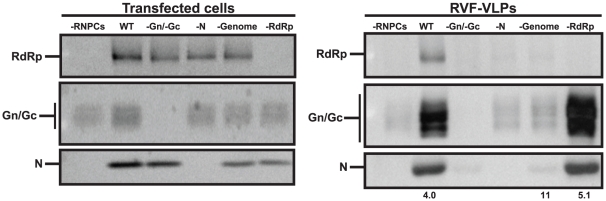
Viral components required for efficient RVF-VLP release. BSR-T7/5 cells were transfected with genome and all of the structural proteins (WT), or one or more of the components was replaced with an equivalent amount of empty vector (-RNPCs, -Gn/Gc, -N, -genome, -RdRp). RNPCs refer to ribonucleoprotein complexes and are defined as genome, N, and RdRp. Transfected cells were analyzed for protein expression by immunoblot. RVF-VLPs were immune precipitated from the clarified media from transfected cells and analyzed by immunoblot. The numbers below the immunoblots indicate the ratio of glycoprotein to N signal.

Gn can package both RdRp and N independently into RVF-VLPs. Therefore, we investigated the individual roles of each of these viral components in the release of RVF-VLPs from cells. The minimal set of viral components necessary for the efficient cellular release of RVF-VLPs was determined by transfection of cells with minigenome, pN, pRdRp, pGn, and pGc, or with one or more of the expression plasmids replaced by an equivalent amount of empty vector. The RVF-VLPs were visualized by transmission electron microscopy, immune precipitated RVF-VLPs were analyzed for protein content by immunoblot, and infectivity of RVF-VLPs was determined by RLuc expression in target cells. When wild-type viral proteins and genome were expressed, RVF-VLPs were released from the cell and all viral proteins could be visualized (Fig. 6). No particles were visualized by electron microscopy when the genome, N, and RdRp, which form the viral ribonucleoprotein complex, were expressed without the envelope glycoproteins (-Gn/-Gc) and there was no expression of the RLuc reporter above background levels (-genome; no luciferase gene) in target cells (Table 4). Our results corroborate the results of previous findings that ribonucleoprotein complexes are not released from the cell in the absence of glycoproteins [24].

**Table 4:** Encapsidated genome required for infectivity.

Sample	AverageLog (RLU/mL)	Std. Dev.Log (RLU/mL)
WT	8.15*	0.471
-Gn/-Gc	4.47	0.515
-N	4.22	0.696
-Genome	3.78	0.741

*Value is significantly different from -Gn/-Gc, p<0.001.

We next determined which viral components are necessary for efficient RVF-VLP release. For the purpose of this analysis we equated RVF-VLP release with Gn/Gc signal on immunoblots of isolated RVF-VLPs. Gn/Gc expression levels were measured and normalized to expression levels in transfected cells. The experimental condition that included all structural proteins and genome (WT) was designated as 100% release efficiency and the condition in which both envelope glycoproteins were omitted from the transfection (-Gn/Gc) was considered background (Fig. 6 and Table 3). The samples lacking N or the genome exhibited average release efficiencies of only 15.6 and 18.1%, respectively (Table 3). These efficiencies were similar to when the entire ribonucleoprotein complex was absent (Fig. 6 and Table 3). Our results demonstrate that efficient release requires both N and the genome, presumably in the form of encapsidated genome. Conversely, the absence of RdRp did not adversely affect the efficiency of release of the glycoproteins or the packaging of N (Fig. 6 and Table 3), indicating that RdRp does not play a critical role in viral budding or release. Particles can be generated at low levels when either Gn or Gc is absent, however the amount of glycoproteins released was at or below the limit of detection by immunoblot. Release efficiencies were decreased ∼2-fold when either GnK48 or GcW1 (Table 3) was expressed, however this decrease was only significant for GnK48. Since N is packaged under both conditions (Fig. 4A and 5A), the cytoplasmic tails of these glycoproteins may perform additional functions in the release process.

### Genome is packaged into RVF-VLPs that lack RdRp

**Figure 7:**
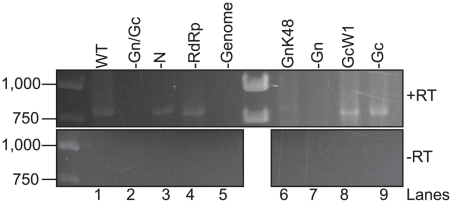
Genomic RNA is packaged into RVF-VLPs that lack RdRp. BSR-T7/5 cells were transfected with genome and all of the structural proteins (WT), or one or more of the components was replaced with an equivalent amount of empty vector (-Gn/Gc, -N, -RdRp, -genome, -Gn and -Gc) or with plasmids expressing mutant alleles of Gn or Gc (GnK48 or GcW1). At 24 h the media was replaced with fresh media containing benzonase nuclease. At 48 h the media was removed, clarified and the RVF-VLPs were harvested by ultracentrifugation. The RNA was isolated from the RVF-VLPs and cDNA was generated with primers that recognize the genomic termini and reverse transcriptase (+RT). Duplicate samples were also run without reverse transcriptase (-RT). PCR was performed using primers that flank the intergenic region. The numbers on the left of the gel image indicate size standards.

The presence of genome in RVF-VLPs can be inferred from RLuc activity in infected cells. However, many of the experimental conditions used in this study (-N, -Gn, -Gc, -RdRp and GnK48) do not produce RLuc in target cells at levels significantly different from a negative control (-Gn/Gc) (Table 1). Since RVFV can package both sense and anti-sense genomic RNA [25], [26], the requisite packaging signals must be present on both senses of genomic RNA. Therefore cells used to make RVF-VLPs in the absence of replication (e.g. –N and –RdRp) contain genomic RNA (synthesized by T7 RNA polymerase) that is competent for packaging. The presence or absence of both senses of genomic RNA in RVF-VLPs was assayed by RT-PCR. BSR-T7/5 cells were transfected with minigenome, pN, pRdRp, pGn, pGc or one or more of the components were replaced with an equivalent amount of empty vector, pGnK48 or pGcW1. At 24 h the media was replaced with fresh media containing benzonase, a nuclease that degrades RNA and DNA. At 48 h RVF-VLPs were harvested and 10% of each sample was passaged onto target cells. At 24 h post-infection the cells were analyzed for RLuc activity (Table 5). The addition of benzonase to the samples substantially reduced the background of our RVF-VLP infectivity assay (compare Tables 1 and 5), suggesting that carry-over plasmid and/or RNA released from dying cells is contributing to the background observed in earlier experiments (Tables 1–3). RVF-VLPs were isolated from the remaining sample by ultracentrifugation and then RNA was extracted. The extracted RNA was then subjected to RT-PCR using primers that flank the intergenic region and are expected to generate a 773-nucleotide product. The S segment is ambisense and produces two mRNAs, both of which lack the intergenic region [27], [28]. Therefore, only genomic RNA (either genome or anti-genome sense) can be amplified using our primer set. We obtained an RT-PCR product of the expected size when all viral components were co-expressed (Fig. 7, lane 1) but not when either genome or both glycoproteins were omitted (Fig. 7, lanes 2 and 5). These results were in agreement with our RLuc activity assay (Table 1) that indicates genomic RNA is delivered to target cells when all viral components are co-expressed (WT) but not when the glycoproteins are absent (-Gn/Gc). When RdRp is absent or GnK48 is expressed, RdRp is not found in RVF-VLPs (Fig. 4A), however we obtain a RT-PCR product of the appropriate size (Fig. 7, lanes 4 and 6), demonstrating that genome is packaged. Our immunoblot analysis of immune precipitated RVF-VLPs (Fig. 4A and 5A), cross-linking (Fig. 5B) and immunofluorescence (Fig. 3B) results show that Gn contains the sequences required for RdRp and N recruitment. Although very few RVF-VLPs are produced when Gn or Gc are absent, we found that genomic RNA was present in samples that lack Gc or express the GcW1 allele (Fig. 7, lanes 8–9) but absent when Gn is not expressed (Fig. 7, lane 7), consistent with our results demonstrating that the Gc cytosolic tail is not required for the generation of infectious particles. Surprisingly, we found that genome was present in samples that lack N (Fig. 7, lane 3) indicating that there may be a binding site for genomic RNA on one of the envelope glycoproteins. Since N can bind RNA non-specifically, this result may explain the specificity for genomic RNA in promoting virus release.

**Table 5:** Benzonase treatment reduces background RLuc activity.

Sample	Untransfected		RdRp/N- transfected	
	Log (Average)	Std. Dev.	Log (Average)	Std. Dev.
WT	5.78*	0.08	7.57*	0.01
-Gn/-Gc	2.94	0.03	3.49	0.59
-N	2.97	0.00	2.87	0.01
-RdRp	2.93	0.02	2.90	0.09
-Genome	2.93	0.01	2.84	0.00
GnK48	2.96	0.00	3.28	0.45
-Gn	2.97	0.03	3.34	0.50
GcW1	3.48	0.22	4.43*	0.11
-Gc	2.97	0.03	3.08	0.27

*Values are significantly different from the negative control (-Genome), p<0.05.

## Discussion

**Figure 8:**
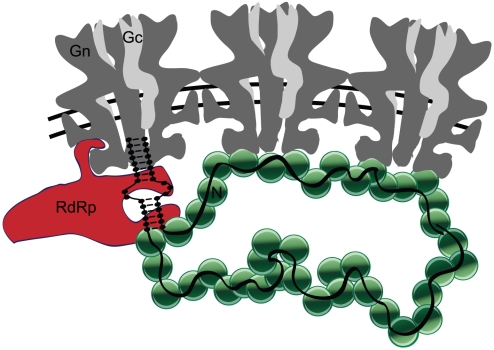
Model for the assembly of RVFV. The diagram represents a slice through the middle of three hexameric capsomers of the virion. Hexameric capsomers contain six copies of heterodimeric Gn (dark gray) and Gc (light gray). Gn is predicted to have two transmembrane domains (TMD). N (green) binds within the first 30 amino acids of cytosolic tail and RdRp (red) binds within the latter 40 amino acids. The genomic segments (black), presumably the terminal regions, bind to the cytosolic tail of Gn. We hypothesize that Gn-N interactions across multiple capsomers induce a membrane curvature that leads to budding of the virion.

Efficient release of RVFV virions requires both the genomic RNA and N, presumably in the form of encapsidated genome. N is a non-specific single-stranded RNA binding protein that undoubtedly binds cellular RNA in the absence of genome [23]. However, genomic RNA is required for efficient release of virus. The requirement for genomic RNA is especially intriguing since it indicates that the genome is not merely a passenger within the virion, but actively participates in release of virus particles. The termini of the genomic segments are complementary and are expected to form a dsRNA structure that contains the sequences necessary for packaging and the promoters for transcription and replication [29]. We hypothesize that the packaging signal is recognized by one of the glycoproteins, most likely Gn (Fig. 8). Viral genomes have not been implicated in stimulating the budding and/or release of any negative or positive-sense RNA virus prior to this report.

Nearly all RVFV particles should contain genome since encapsidated genome acts as the stimulus for virion formation. It is thus reasonable to expect most virus particles to be infectious. Many enveloped RNA viruses yield particle-to-plaque-forming unit (pfu) ratios in the tens or hundreds [30], [31], [32], [33], however studies with Bunyamwera virus (*Orthobunyavirus* genus) determined that the particle-to-pfu ratio approaches one [34]. We propose that the interactions between the encapsidated genomic segments and multiple Gn cause a change in membrane curvature that leads to virus particle budding into the Golgi lumen (Fig. 8). The virus buds when the critical quantity of genome is bound, presumably corresponding to at least one copy each of S, M and L segments. It remains to be seen if the segments are specifically or randomly packaged.

Previous studies with the positive-sense RNA viruses, poliovirus (*Picornaviridae* family) and Flock house virus (*Nodaviridae* family) found that only actively replicating genomes were recruited for virus assembly [35], [36], [37]. For poliovirus, it is hypothesized that translation is coupled to replication of the genome and assembly of the virus, so that only genomes that encode functional proteins are replicated and packaged [35]. For the Flock house virus, it is hypothesized that the replicating and non-replicating RNA genomes segregate to distinct sub-cellular locations, allowing for packaging of only the replicating RNA [37]. In contrast to poliovirus and Flock house virus, RVFV can package replicating or non-replicating ribonucleoprotein complexes. In fact, RVF-VLPs produced in the absence of RdRp contain genome and are released as efficiently as WT RVF-VLPs.

Our results support studies performed by Liu et. al. [38] using a baculovirus expression system for generation of RVF-VLPs in insect cells. They found that particles could be generated through expression of Gn and/or Gc with N. Similarly, we could not identify any particles by electron microscopy unless N and Gn or Gc were co-expressed. Based on their ability to visualize particles, Liu et. al. concluded that only N and the envelope glycoproteins were required for generation of particles, however efficiency of release could not be determined using their methods [38]. We observed particles that lacked genome, however RVF-VLP release was only efficient when genome was present (Table 3).

Most RNA viruses require a matrix protein for the packaging of the ribonucleoprotein complexes and release of viral particles [1], [39], [40], [41], [42], [43], [44], [45], [46], [47], [48], [49], [50], [51], [52], however viruses of the *Bunyaviridae* family do not encode a matrix protein. Based on our results, the Gn cytoplasmic tail appears to function in place of matrix and recruits RdRp, N and possibly, genomic RNA (Fig. 8) into virions. By contrast, the cytosolic portion of Gc was dispensable for recruitment and packaging of RdRp, N and genome. Particles lacking N are inefficiently produced (∼16% of WT levels) however, we were able to confirm that they contain genomic RNA. Although N is capable of non-specifically binding cellular RNA [23], efficient RVF-VLP release requires genomic RNA. These data suggest that genomic RNA is recognized specifically, possibly by Gn, since particles lacking the cytoplasmic portion of Gc (GcW1) are infectious and efficiently produced (∼57% of WT levels).

Different regions of the Gn cytoplasmic tail are required for independent interactions with RdRp and N (Fig. 8). The truncated Gn allele, GnK48, allowed us to define the sequences required for N and RdRp recruitment. The sequence on the Gn cytosolic tail required for interaction with N is located within the first 30 amino acids while that of the RdRp is in the last 40 amino acids. The Gn domain required for N interaction corresponds to a region that is highly hydrophobic. The hydrophobic character of this domain is conserved amongst phleboviruses [12]. Binding of N and RdRp to Gn can occur independently. This observation may reflect the fact that there are few copies of RdRp and many copies of N within a virion. Thus, you would not expect that their binding to Gn would be mutually dependent.

Studies performed with the Uukuniemi virus (*Phlebovirus* genus) found that the Gn cytoplasmic tail is required for the packaging of N, but identified a different region as important for this interaction [53]. The envelope glycoproteins and N of Uukuniemi virus are divergent from the rest of the phlebovirus genus, which may explain why our results contrast. Gn interaction with N is unlikely to be conserved across the five genera within family *Bunyaviridae*, as the envelope glycoproteins and N are not similar. The N (and RdRp) of the hantaviruses independently localize to perinuclear membrane structures when expressed alone, suggesting a distinct mode of assembly [20], [54]. For tospoviruses, independent interactions between Gn and Gc with N were discovered, indicating a possible requirement for both glycoproteins during recruitment [17].

We found no role for Gc in recruitment of N, genome and RdRp, however Gc is necessary for optimal Gn expression, efficient production of RVF-VLPs and possibly, infectivity. Studies performed on RVFV by Besselaar and Blackburn [55] suggest a requirement for Gc in virus entry, as they were able to neutralize virus with antibodies recognizing Gc, either pre- or post- virus absorption. Computational studies have predicted RVFV Gc to be a class II viral fusion protein [56], and previous experiments with other viruses of the *Bunyaviridae* family support Gc being the main determinant of cell fusion [57], [58], [59]. Fusion assays utilizing Gn and Gc of Bunyamwera virus (*Orthobunyavirus* genus, *Bunyaviridae* family) found that deletions in Gc prevented syncytia formation [59]. Additional experiments with La Crosse and Tahyna viruses (*Orthobunyavirus* genus) identified Gc as a fusion protein using chimeras, site-directed mutagenesis, and cell-cell-fusion assays [57], [58], [59].

Although it has been widely acknowledged that the RdRp is fundamental to replication and transcription of the RNA virus genome, other roles for the RVFV RdRp have not been previously explored. We found that RdRp was not required for the efficient cellular release of virus or packaging of N and genome. However, RVF-VLPs that lack RdRp, or express a catalytically inactive RdRp, cannot be complemented in *trans*. Complementing in *trans* with viral components required for transcription/replication is not unprecedented. Studies with the Ebola virus (*Ebolavirus* genus, *Filoviridae* family), which is a non-segmented negative-sense RNA virus, investigated the viral components necessary for the generation of infectious particles. The Ebola virus VP30 protein, which is required for replication/transcription by the RdRp, could be complemented in *trans* for restoration of activity in Ebola-VLP-infected target cells [60]. Recently it was discovered that *trans* expressed influenza virus (*Orthomyxoviridae* family) RdRp can replicate viral ribonucleoproteins (vRNPs) and become incorporated into progeny vRNPs [61], however only *cis* RdRp could transcribe vRNPs. This result suggests that the *cis* (packaged) RdRp is somehow different from the *trans* (cellular) RdRp [61]. Our complementation studies suggest that a similar phenomenon may be occurring with RVFV RdRp, such that a catalytically active RdRp must be packaged in order for *trans* expressed RdRp to transcribe a reporter gene.

We have illuminated roles for each of the viral components in the assembly, cellular release, and infectivity of RVFV. The interaction of genome and N with Gn triggers release of virus. Our results illustrate a novel mechanism for the efficient generation of infectious virus particles. The design and screening of therapeutics targeting the Gn cytoplasmic tail may offer a novel target for inhibition of both virus release and packaging of the RdRp and encapsidated genome.
